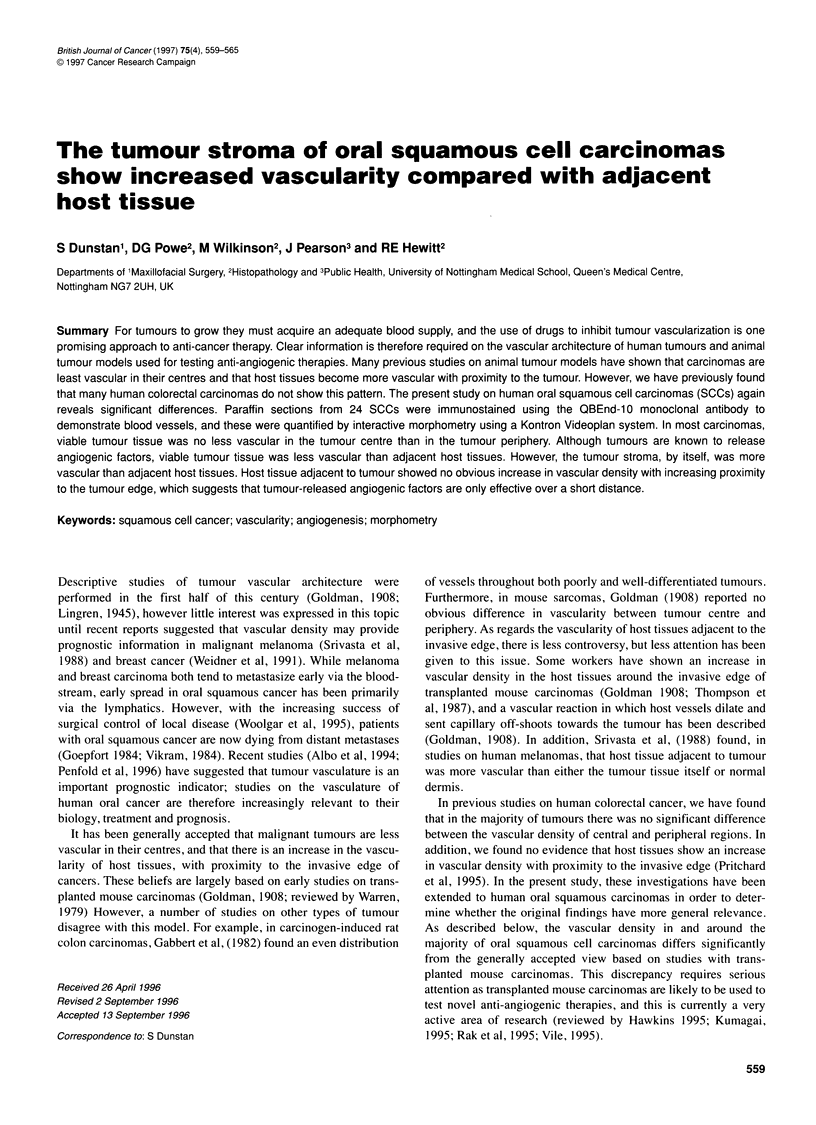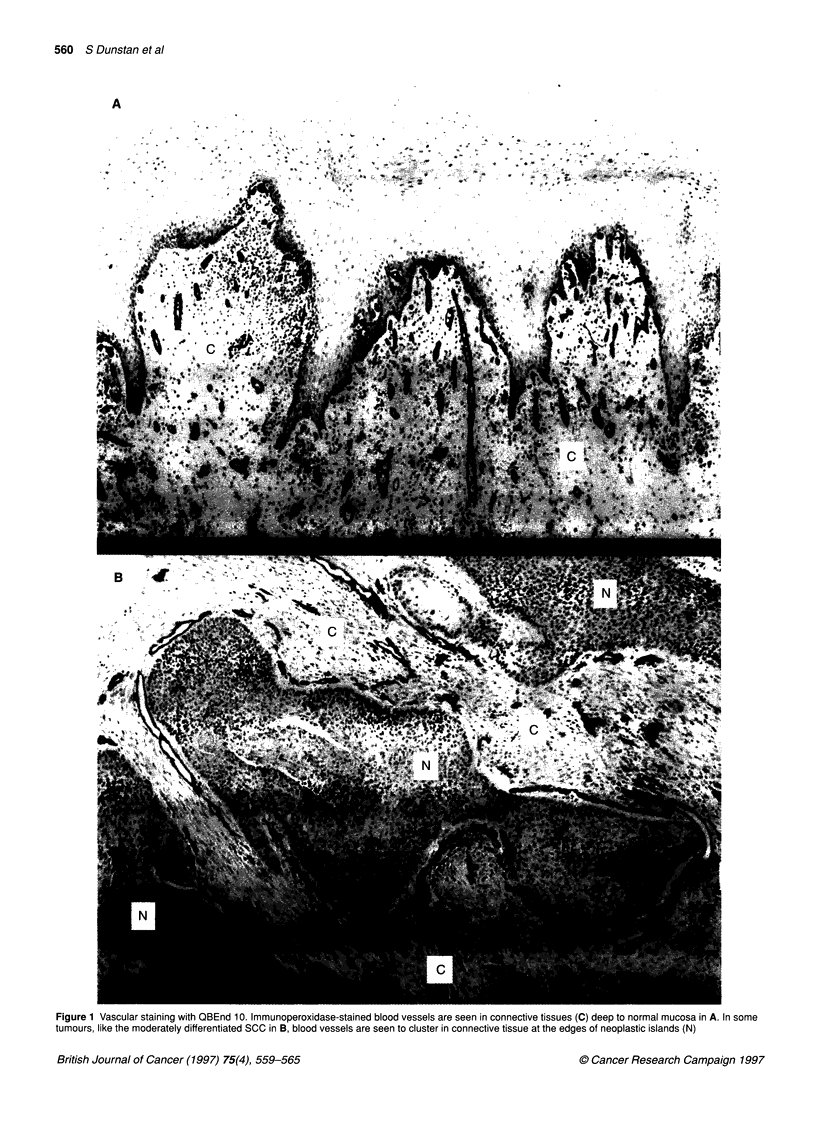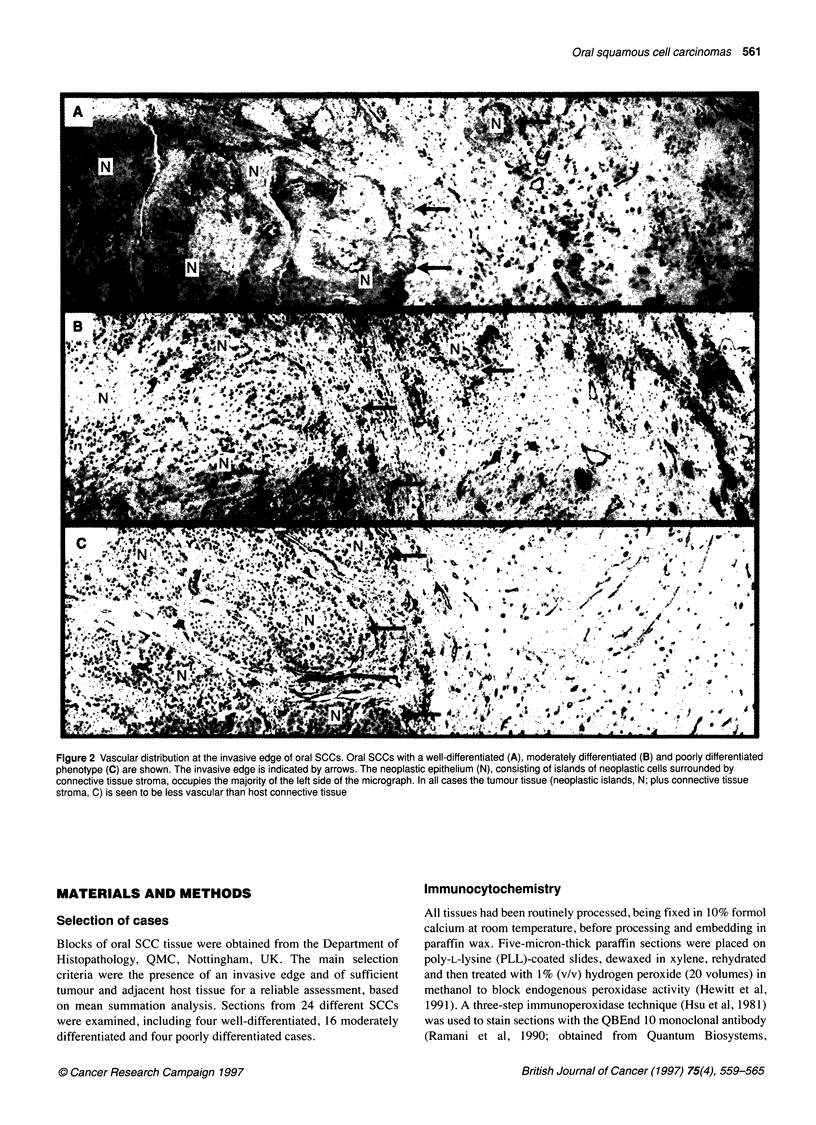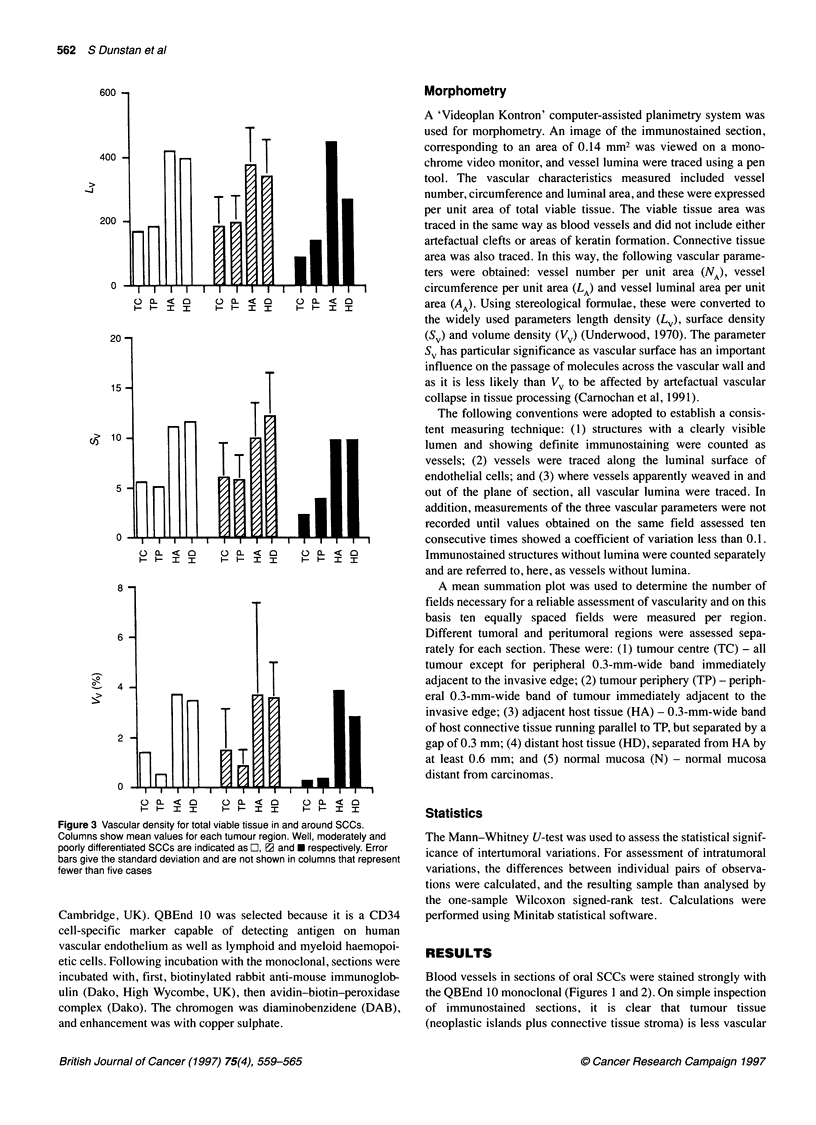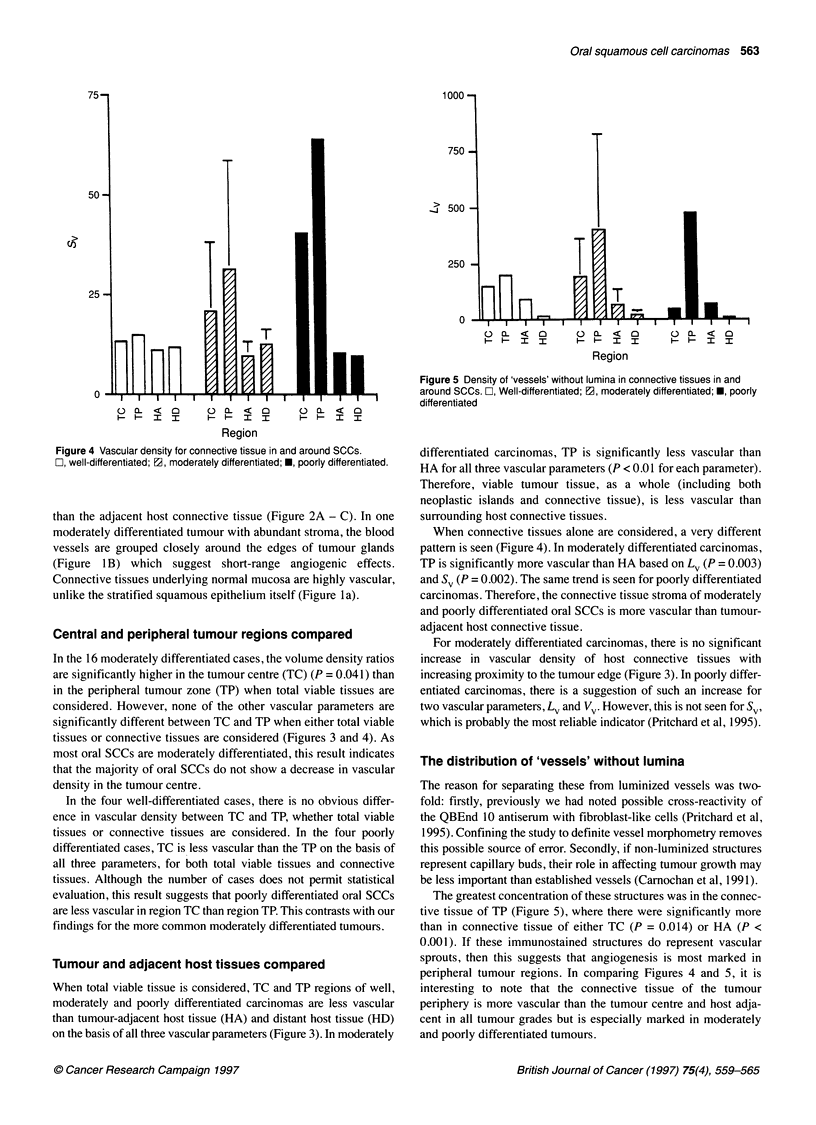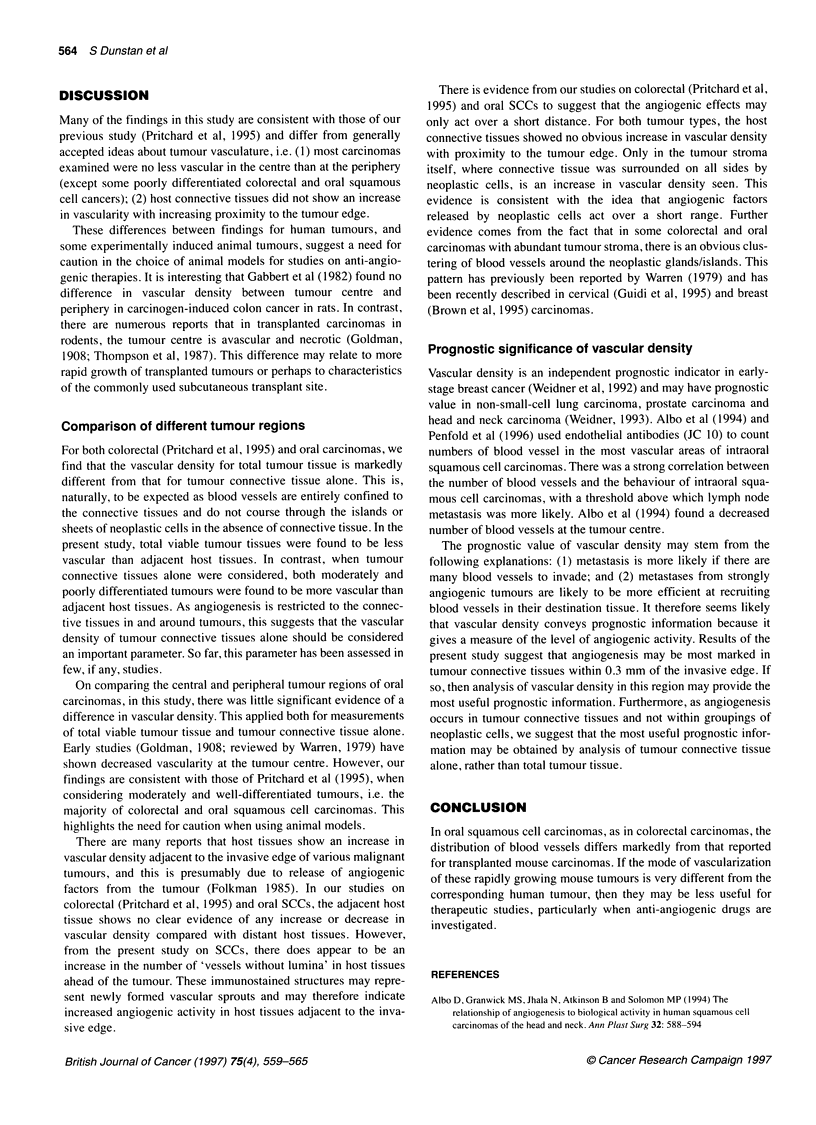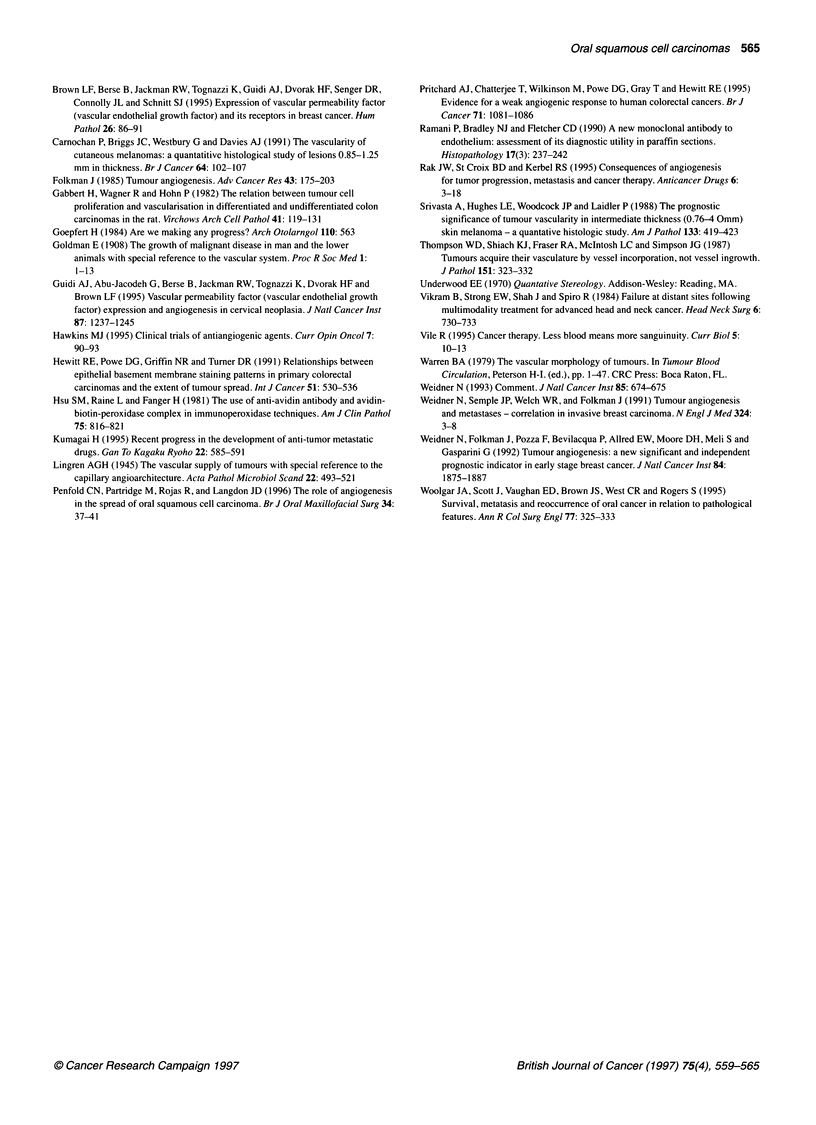# The tumour stroma of oral squamous cell carcinomas show increased vascularity compared with adjacent host tissue.

**DOI:** 10.1038/bjc.1997.98

**Published:** 1997

**Authors:** S. Dunstan, D. G. Powe, M. Wilkinson, J. Pearson, R. E. Hewitt

**Affiliations:** Department of Maxillofacial Surgery, University of Nottingham Medical School, Queen's Medical Centre, UK.

## Abstract

**Images:**


British Journal of Cancer (1997) 75(4), 559–565
© 1997 Cancer Research Campaign

### The tumour stroma of oral squamous cell carcinomas show increased vascularity compared with adjacent host tissue

**S Dunstan[1], DG Powe[2], M Wilkinson[2], J Pearson[3] and RE Hewitt[2]**

Departments of [1]Maxillofacial Surgery, [2]Histopathology and [3]Public Health, University of Nottingham Medical School, Queen's Medical Centre, Nottingham NG7 2UH, UK

**Summary** For tumours to grow they must acquire an adequate blood supply, and the use of drugs to inhibit tumour vascularization is one promising approach to anti-cancer therapy. Clear information is therefore required on the vascular architecture of human tumours and animal tumour models used for testing anti-angiogenic therapies. Many previous studies on animal tumour models have shown that carcinomas are least vascular in their centres and that host tissues become more vascular with proximity to the tumour. However, we have previously found that many human colorectal carcinomas do not show this pattern. The present study on human oral squamous cell carcinomas (SCCs) again reveals significant differences. Paraffin sections from 24 SCCs were immunostained using the QBEnd-10 monoclonal antibody to demonstrate blood vessels, and these were quantified by interactive morphometry using a Kontron Videoplan system. In most carcinomas, viable tumour tissue was no less vascular in the tumour centre than in the tumour periphery. Although tumours are known to release angiogenic factors, viable tumour tissue was less vascular than adjacent host tissues. However, the tumour stroma, by itself, was more vascular than adjacent host tissues. Host tissue adjacent to tumour showed no obvious increase in vascular density with increasing proximity to the tumour edge, which suggests that tumour-released angiogenic factors are only effective over a short distance.

**Keywords:** squamous cell cancer; vascularity; angiogenesis; morphometry

Descriptive studies of tumour vascular architecture were performed in the first half of this century (Goldman, 1908; Lingren, 1945), however little interest was expressed in this topic until recent reports suggested that vascular density may provide prognostic information in malignant melanoma (Srivasta et al, 1988) and breast cancer (Weidner et al, 1991). While melanoma and breast carcinoma both tend to metastasize early via the blood-stream, early spread in oral squamous cancer has been primarily via the lymphatics. However, with the increasing success of surgical control of local disease (Woolgar et al, 1995), patients with oral squamous cancer are now dying from distant metastases (Goepfort 1984; Vikram, 1984). Recent studies (Albo et al, 1994; Penfold et al, 1996) have suggested that tumour vasculature is an important prognostic indicator; studies on the vasculature of human oral cancer are therefore increasingly relevant to their biology, treatment and prognosis.

It has been generally accepted that malignant tumours are less vascular in their centres, and that there is an increase in the vascularity of host tissues, with proximity to the invasive edge of cancers. These beliefs are largely based on early studies on transplanted mouse carcinomas (Goldman, 1908; reviewed by Warren, 1979) However, a number of studies on other types of tumour disagree with this model. For example, in carcinogen-induced rat colon carcinomas, Gabbert et al, (1982) found an even distribution of vessels throughout both poorly and well-differentiated tumours. Furthermore, in mouse sarcomas, Goldman (1908) reported no obvious difference in vascularity between tumour centre and periphery. As regards the vascularity of host tissues adjacent to the invasive edge, there is less controversy, but less attention has been given to this issue. Some workers have shown an increase in vascular density in the host tissues around the invasive edge of transplanted mouse carcinomas (Goldman 1908; Thompson et al, 1987), and a vascular reaction in which host vessels dilate and sent capillary off-shoots towards the tumour has been described (Goldman, 1908). In addition, Srivasta et al, (1988) found, in studies on human melanomas, that host tissue adjacent to tumour was more vascular than either the tumour tissue itself or normal dermis.

In previous studies on human colorectal cancer, we have found that in the majority of tumours there was no significant difference between the vascular density of central and peripheral regions. In addition, we found no evidence that host tissues show an increase in vascular density with proximity to the invasive edge (Pritchard et al, 1995). In the present study, these investigations have been extended to human oral squamous carcinomas in order to determine whether the original findings have more general relevance. As described below, the vascular density in and around the majority of oral squamous cell carcinomas differs significantly from the generally accepted view based on studies with transplanted mouse carcinomas. This discrepancy requires serious attention as transplanted mouse carcinomas are likely to be used to test novel anti-angiogenic therapies, and this is currently a very active area of research (reviewed by Hawkins 1995; Kumagai, 1995; Rak et al, 1995; Vile, 1995).

*Received 26 April 1996*
*Revised 2 September 1996*
*Accepted 13 September 1996*

*Correspondence to*: S Dunstan

**Figure 1** Vascular staining with QBEnd 10. Immunoperoxidase-stained blood vessels are seen in connective tissues (**C**) deep to normal mucosa in **A**. In some tumours, like the moderately differentiated SCC in **B**, blood vessels are seen to cluster in connective tissue at the edges of neoplastic islands (N)

© Cancer Research Campaign 1997

**Figure 2** Vascular distribution at the invasive edge of oral SCCs. Oral SCCs with a well-differentiated (**A**), moderately differentiated (**B**) and poorly differentiated phenotype (**C**) are shown. The invasive edge is indicated by arrows. The neoplastic epithelium (N), consisting of islands of neoplastic cells surrounded by connective tissue stroma, occupies the majority of the left side of the micrograph. In all cases the tumour tissue (neoplastic islands, N; plus connective tissue stroma, C) is seen to be less vascular than host connective tissue

### MATERIALS AND METHODS

### Selection of cases

Blocks of oral SCC tissue were obtained from the Department of Histopathology, QMC, Nottingham, UK. The main selection criteria were the presence of an invasive edge and of sufficient tumour and adjacent host tissue for a reliable assessment, based on mean summation analysis. Sections from 24 different SCCs were examined, including four well-differentiated, 16 moderately differentiated and four poorly differentiated cases.

### Immunocytochemistry

All tissues had been routinely processed, being fixed in 10% formol calcium at room temperature, before processing and embedding in paraffin wax. Five-micron-thick paraffin sections were placed on poly-L-lysine (PLL)-coated slides, dewaxed in xylene, rehydrated and then treated with 1% (v/v) hydrogen peroxide (20 volumes) in methanol to block endogenous peroxidase activity (Hewitt et al, 1991). A three-step immunoperoxidase technique (Hsu et al, 1981) was used to stain sections with the QBEnd 10 monoclonal antibody (Ramani et al, 1990; obtained from Quantum Biosystems,

© Cancer Research Campaign 1997

**Figure 3** Vascular density for total viable tissue in and around SCCs. Columns show mean values for each tumour region. Well, moderately and poorly differentiated SCCs are indicated as □, ▨ and ■ respectively. Error bars give the standard deviation and are not shown in columns that represent fewer than five cases

Cambridge, UK). QBEnd 10 was selected because it is a CD34 cell-specific marker capable of detecting antigen on human vascular endothelium as well as lymphoid and myeloid haemopoietic cells. Following incubation with the monoclonal, sections were incubated with, first, biotinylated rabbit anti-mouse immunoglobulin (Dako, High Wycombe, UK), then avidin–biotin–peroxidase complex (Dako). The chromogen was diaminobenzidene (DAB), and enhancement was with copper sulphate.

### Morphometry

A 'Videoplan Kontron' computer-assisted planimetry system was used for morphometry. An image of the immunostained section, corresponding to an area of 0.14 $mm^2$ was viewed on a monochrome video monitor, and vessel lumina were traced using a pen tool. The vascular characteristics measured included vessel number, circumference and luminal area, and these were expressed per unit area of total viable tissue. The viable tissue area was traced in the same way as blood vessels and did not include either artefactual clefts or areas of keratin formation. Connective tissue area was also traced. In this way, the following vascular parameters were obtained: vessel number per unit area ($N_A$), vessel circumference per unit area ($L_A$) and vessel luminal area per unit area ($A_A$). Using stereological formulae, these were converted to the widely used parameters length density ($L_V$), surface density ($S_V$) and volume density ($V_V$) (Underwood, 1970). The parameter $S_V$ has particular significance as vascular surface has an important influence on the passage of molecules across the vascular wall and as it is less likely than $V_V$ to be affected by artefactual vascular collapse in tissue processing (Carnochan et al, 1991).

The following conventions were adopted to establish a consistent measuring technique: (1) structures with a clearly visible lumen and showing definite immunostaining were counted as vessels; (2) vessels were traced along the luminal surface of endothelial cells; and (3) where vessels apparently weaved in and out of the plane of section, all vascular lumina were traced. In addition, measurements of the three vascular parameters were not recorded until values obtained on the same field assessed ten consecutive times showed a coefficient of variation less than 0.1. Immunostained structures without lumina were counted separately and are referred to, here, as vessels without lumina.

A mean summation plot was used to determine the number of fields necessary for a reliable assessment of vascularity and on this basis ten equally spaced fields were measured per region. Different tumoral and peritumoral regions were assessed separately for each section. These were: (1) tumour centre (TC) – all tumour except for peripheral 0.3-mm-wide band immediately adjacent to the invasive edge; (2) tumour periphery (TP) – peripheral 0.3-mm-wide band of tumour immediately adjacent to the invasive edge; (3) adjacent host tissue (HA) – 0.3-mm-wide band of host connective tissue running parallel to TP, but separated by a gap of 0.3 mm; (4) distant host tissue (HD), separated from HA by at least 0.6 mm; and (5) normal mucosa (N) – normal mucosa distant from carcinomas.

### Statistics

The Mann–Whitney $U$-test was used to assess the statistical significance of intertumoral variations. For assessment of intratumoral variations, the differences between individual pairs of observations were calculated, and the resulting sample than analysed by the one-sample Wilcoxon signed-rank test. Calculations were performed using Minitab statistical software.

### RESULTS

Blood vessels in sections of oral SCCs were stained strongly with the QBEnd 10 monoclonal (Figures 1 and 2). On simple inspection of immunostained sections, it is clear that tumour tissue (neoplastic islands plus connective tissue stroma) is less vascular

© Cancer Research Campaign 1997

**Figure 4** Vascular density for connective tissue in and around SCCs. □, well-differentiated; ▨, moderately differentiated; ■, poorly differentiated.

**Figure 5** Density of 'vessels' without lumina in connective tissues in and around SCCs. □, Well-differentiated; ▨, moderately differentiated; ■, poorly differentiated than the adjacent host connective tissue (Figure 2A – C). In one moderately differentiated tumour with abundant stroma, the blood vessels are grouped closely around the edges of tumour glands (Figure 1B) which suggest short-range angiogenic effects. Connective tissues underlying normal mucosa are highly vascular, unlike the stratified squamous epithelium itself (Figure 1a).

### Central and peripheral tumour regions compared

In the 16 moderately differentiated cases, the volume density ratios are significantly higher in the tumour centre (TC) ($P = 0.041$) than in the peripheral tumour zone (TP) when total viable tissues are considered. However, none of the other vascular parameters are significantly different between TC and TP when either total viable tissues or connective tissues are considered (Figures 3 and 4). As most oral SCCs are moderately differentiated, this result indicates that the majority of oral SCCs do not show a decrease in vascular density in the tumour centre.

In the four well-differentiated cases, there is no obvious difference in vascular density between TC and TP, whether total viable tissues or connective tissues are considered. In the four poorly differentiated cases, TC is less vascular than the TP on the basis of all three parameters, for both total viable tissues and connective tissues. Although the number of cases does not permit statistical evaluation, this result suggests that poorly differentiated oral SCCs are less vascular in region TC than region TP. This contrasts with our findings for the more common moderately differentiated tumours.

### Tumour and adjacent host tissues compared

When total viable tissue is considered, TC and TP regions of well, moderately and poorly differentiated carcinomas are less vascular than tumour-adjacent host tissue (HA) and distant host tissue (HD) on the basis of all three vascular parameters (Figure 3). In moderately differentiated carcinomas, TP is significantly less vascular than HA for all three vascular parameters ($P < 0.01$ for each parameter). Therefore, viable tumour tissue, as a whole (including both neoplastic islands and connective tissue), is less vascular than surrounding host connective tissues.

When connective tissues alone are considered, a very different pattern is seen (Figure 4). In moderately differentiated carcinomas, TP is significantly more vascular than HA based on $L_V$ ($P = 0.003$) and $S_V$ ($P = 0.002$). The same trend is seen for poorly differentiated carcinomas. Therefore, the connective tissue stroma of moderately and poorly differentiated oral SCCs is more vascular than tumour-adjacent host connective tissue.

For moderately differentiated carcinomas, there is no significant increase in vascular density of host connective tissues with increasing proximity to the tumour edge (Figure 3). In poorly differentiated carcinomas, there is a suggestion of such an increase for two vascular parameters, $L_V$ and $V_V$. However, this is not seen for $S_V$, which is probably the most reliable indicator (Pritchard et al, 1995).

### The distribution of 'vessels' without lumina

The reason for separating these from luminized vessels was twofold: firstly, previously we had noted possible cross-reactivity of the QBEnd 10 antiserum with fibroblast-like cells (Pritchard et al, 1995). Confining the study to definite vessel morphometry removes this possible source of error. Secondly, if non-luminized structures represent capillary buds, their role in affecting tumour growth may be less important than established vessels (Carnochan et al, 1991).

The greatest concentration of these structures was in the connective tissue of TP (Figure 5), where there were significantly more than in connective tissue of either TC ($P = 0.014$) or HA ($P < 0.001$). If these immunostained structures do represent vascular sprouts, then this suggests that angiogenesis is most marked in peripheral tumour regions. In comparing Figures 4 and 5, it is interesting to note that the connective tissue of the tumour periphery is more vascular than the tumour centre and host adjacent in all tumour grades but is especially marked in moderately and poorly differentiated tumours.

© Cancer Research Campaign 1997

### DISCUSSION

Many of the findings in this study are consistent with those of our previous study (Pritchard et al, 1995) and differ from generally accepted ideas about tumour vasculature, i.e. (1) most carcinomas examined were no less vascular in the centre than at the periphery (except some poorly differentiated colorectal and oral squamous cell cancers); (2) host connective tissues did not show an increase in vascularity with increasing proximity to the tumour edge.

These differences between findings for human tumours, and some experimentally induced animal tumours, suggest a need for caution in the choice of animal models for studies on anti-angiogenic therapies. It is interesting that Gabbert et al (1982) found no difference in vascular density between tumour centre and periphery in carcinogen-induced colon cancer in rats. In contrast, there are numerous reports that in transplanted carcinomas in rodents, the tumour centre is avascular and necrotic (Goldman, 1908; Thompson et al, 1987). This difference may relate to more rapid growth of transplanted tumours or perhaps to characteristics of the commonly used subcutaneous transplant site.

### Comparison of different tumour regions

For both colorectal (Pritchard et al, 1995) and oral carcinomas, we find that the vascular density for total tumour tissue is markedly different from that for tumour connective tissue alone. This is, naturally, to be expected as blood vessels are entirely confined to the connective tissues and do not course through the islands or sheets of neoplastic cells in the absence of connective tissue. In the present study, total viable tumour tissues were found to be less vascular than adjacent host tissues. In contrast, when tumour connective tissues alone were considered, both moderately and poorly differentiated tumours were found to be more vascular than adjacent host tissues. As angiogenesis is restricted to the connective tissues in and around tumours, this suggests that the vascular density of tumour connective tissues alone should be considered an important parameter. So far, this parameter has been assessed in few, if any, studies.

On comparing the central and peripheral tumour regions of oral carcinomas, in this study, there was little significant evidence of a difference in vascular density. This applied both for measurements of total viable tumour tissue and tumour connective tissue alone. Early studies (Goldman, 1908; reviewed by Warren, 1979) have shown decreased vascularity at the tumour centre. However, our findings are consistent with those of Pritchard et al (1995), when considering moderately and well-differentiated tumours, i.e. the majority of colorectal and oral squamous cell carcinomas. This highlights the need for caution when using animal models.

There are many reports that host tissues show an increase in vascular density adjacent to the invasive edge of various malignant tumours, and this is presumably due to release of angiogenic factors from the tumour (Folkman 1985). In our studies on colorectal (Pritchard et al, 1995) and oral SCCs, the adjacent host tissue shows no clear evidence of any increase or decrease in vascular density compared with distant host tissues. However, from the present study on SCCs, there does appear to be an increase in the number of 'vessels without lumina' in host tissues ahead of the tumour. These immunostained structures may represent newly formed vascular sprouts and may therefore indicate increased angiogenic activity in host tissues adjacent to the invasive edge.

There is evidence from our studies on colorectal (Pritchard et al, 1995) and oral SCCs to suggest that the angiogenic effects may only act over a short distance. For both tumour types, the host connective tissues showed no obvious increase in vascular density with proximity to the tumour edge. Only in the tumour stroma itself, where connective tissue was surrounded on all sides by neoplastic cells, is an increase in vascular density seen. This evidence is consistent with the idea that angiogenic factors released by neoplastic cells act over a short range. Further evidence comes from the fact that in some colorectal and oral carcinomas with abundant tumour stroma, there is an obvious clustering of blood vessels around the neoplastic glands/islands. This pattern has previously been reported by Warren (1979) and has been recently described in cervical (Guidi et al, 1995) and breast (Brown et al, 1995) carcinomas.

### Prognostic significance of vascular density

Vascular density is an independent prognostic indicator in early-stage breast cancer (Weidner et al, 1992) and may have prognostic value in non-small-cell lung carcinoma, prostate carcinoma and head and neck carcinoma (Weidner, 1993). Albo et al (1994) and Penfold et al (1996) used endothelial antibodies (JC 10) to count numbers of blood vessel in the most vascular areas of intraoral squamous cell carcinomas. There was a strong correlation between the number of blood vessels and the behaviour of intraoral squamous cell carcinomas, with a threshold above which lymph node metastasis was more likely. Albo et al (1994) found a decreased number of blood vessels at the tumour centre.

The prognostic value of vascular density may stem from the following explanations: (1) metastasis is more likely if there are many blood vessels to invade; and (2) metastases from strongly angiogenic tumours are likely to be more efficient at recruiting blood vessels in their destination tissue. It therefore seems likely that vascular density conveys prognostic information because it gives a measure of the level of angiogenic activity. Results of the present study suggest that angiogenesis may be most marked in tumour connective tissues within 0.3 mm of the invasive edge. If so, then analysis of vascular density in this region may provide the most useful prognostic information. Furthermore, as angiogenesis occurs in tumour connective tissues and not within groupings of neoplastic cells, we suggest that the most useful prognostic information may be obtained by analysis of tumour connective tissue alone, rather than total tumour tissue.

### CONCLUSION

In oral squamous cell carcinomas, as in colorectal carcinomas, the distribution of blood vessels differs markedly from that reported for transplanted mouse carcinomas. If the mode of vascularization of these rapidly growing mouse tumours is very different from the corresponding human tumour, then they may be less useful for therapeutic studies, particularly when anti-angiogenic drugs are investigated.

### REFERENCES

Albo D, Granwick MS, Jhala N, Atkinson B and Solomon MP (1994) The relationship of angiogenesis to biological activity in human squamous cell carcinomas of the head and neck. *Ann Plast Surg* **32**: 588–594

*© Cancer Research Campaign 1997*

Brown LF, Berse B, Jackman RW, Tognazzi K, Guidi AJ, Dvorak HF, Senger DR, Connolly JL and Schnitt SJ (1995) Expression of vascular permeability factor (vascular endothelial growth factor) and its receptors in breast cancer. *Hum Pathol* **26**: 86–91

Carnochan P, Briggs JC, Westbury G and Davies AJ (1991) The vascularity of cutaneous melanomas: a quantatitive histological study of lesions 0.85–1.25 mm in thickness. *Br J Cancer* **64**: 102–107

Folkman J (1985) Tumour angiogenesis. *Adv Cancer Res* **43**: 175–203

Gabbert H, Wagner R and Hohn P (1982) The relation between tumour cell proliferation and vascularisation in differentiated and undifferentiated colon carcinomas in the rat. *Virchows Arch Cell Pathol* **41**: 119–131

Goepfert H (1984) Are we making any progress? *Arch Otolarngol* **110**: 563

Goldman E (1908) The growth of malignant disease in man and the lower animals with special reference to the vascular system. *Proc R Soc Med* **1**: 1–13

Guidi AJ, Abu-Jacodeh G, Berse B, Jackman RW, Tognazzi K, Dvorak HF and Brown LF (1995) Vascular permeability factor (vascular endothelial growth factor) expression and angiogenesis in cervical neoplasia. *J Natl Cancer Inst* **87**: 1237–1245

Hawkins MJ (1995) Clinical trials of antiangiogenic agents. *Curr Opin Oncol* **7**: 90–93

Hewitt RE, Powe DG, Griffin NR and Turner DR (1991) Relationships between epithelial basement membrane staining patterns in primary colorectal carcinomas and the extent of tumour spread. *Int J Cancer* **51**: 530–536

Hsu SM, Raine L and Fanger H (1981) The use of anti-avidin antibody and avidin-biotin-peroxidase complex in immunoperoxidase techniques. *Am J Clin Pathol* **75**: 816–821

Kumagai H (1995) Recent progress in the development of anti-tumor metastatic drugs. *Gan To Kagaku Ryoho* **22**: 585–591

Lingren AGH (1945) The vascular supply of tumours with special reference to the capillary angioarchitecture. *Acta Pathol Microbiol Scand* **22**: 493–521

Penfold CN, Partridge M, Rojas R, and Langdon JD (1996) The role of angiogenesis in the spread of oral squamous cell carcinoma. *Br J Oral Maxillofacial Surg* **34**: 37–41

Pritchard AJ, Chatterjee T, Wilkinson M, Powe DG, Gray T and Hewitt RE (1995) Evidence for a weak angiogenic response to human colorectal cancers. *Br J Cancer* **71**: 1081–1086

Ramani P, Bradley NJ and Fletcher CD (1990) A new monoclonal antibody to endothelium: assessment of its diagnostic utility in paraffin sections. *Histopathology* **17**(3): 237–242

Rak JW, St Croix BD and Kerbel RS (1995) Consequences of angiogenesis for tumor progression, metastasis and cancer therapy. *Anticancer Drugs* **6**: 3–18

Srivasta A, Hughes LE, Woodcock JP and Laidler P (1988) The prognostic significance of tumour vascularity in intermediate thickness (0.76–4 Omm) skin melanoma – a quantative histologic study. *Am J Pathol* **133**: 419–423

Thompson WD, Shiach KJ, Fraser RA, McIntosh LC and Simpson JG (1987) Tumours acquire their vasculature by vessel incorporation, not vessel ingrowth. *J Pathol* **151**: 323–332

Underwood EE (1970) *Quantative Stereology*. Addison-Wesley: Reading, MA.

Vikram B, Strong EW, Shah J and Spiro R (1984) Failure at distant sites following multimodality treatment for advanced head and neck cancer. *Head Neck Surg* **6**: 730–733

Vile R (1995) Cancer therapy. Less blood means more sanguinuity. *Curr Biol* **5**: 10–13

Warren BA (1979) The vascular morphology of tumours. In *Tumour Blood Circulation*, Peterson H-I. (ed.), pp. 1–47. CRC Press: Boca Raton, FL.

Weidner N (1993) Comment. *J Natl Cancer Inst* **85**: 674–675

Weidner N, Semple JP, Welch WR, and Folkman J (1991) Tumour angiogenesis and metastases – correlation in invasive breast carcinoma. *N Engl J Med* **324**: 3–8

Weidner N, Folkman J, Pozza F, Bevilacqua P, Allred EW, Moore DH, Meli S and Gasparini G (1992) Tumour angiogenesis: a new significant and independent prognostic indicator in early stage breast cancer. *J Natl Cancer Inst* **84**: 1875–1887

Woolgar JA, Scott J, Vaughan ED, Brown JS, West CR and Rogers S (1995) Survival, metatasis and reoccurrence of oral cancer in relation to pathological features. *Ann R Col Surg Engl* **77**: 325–333

© Cancer Research Campaign 1997